# Efficacy and safety of iclepertin (BI 425809) in patients with schizophrenia: CONNEX, a Phase III randomised controlled trial programme

**DOI:** 10.1192/j.eurpsy.2023.1325

**Published:** 2023-07-19

**Authors:** P. Falkai, C. Reuteman-Fowler, Z. Blahova, S. Ikezawa, S. R. Marder, J. H. Krystal

**Affiliations:** 1 Clinic of Psychiatry and Psychotherapy, Ludwig Maximilians University Munich, Munich, Germany; 2 Boehringer Ingelheim Pharmaceuticals Inc, Ridgefield, United States; 3 Boehringer Ingelheim RCV GmbH & Co. KG, Vienna, Austria; 4 Graduate School of Arts and Sciences, The University of Tokyo, Tokyo, Japan; 5 Department of Psychiatry and Behavioral Sciences, David Geffen School of Medicine, Los Angeles; 6 Department of Psychiatry, Yale University School of Medicine, New Haven, United States

## Abstract

**Introduction:**

Cognitive impairment (CI) is a major determinant of poor functional outcome in schizophrenia and there are currently no available pharmacotherapies. Deficits in glutamatergic signalling play a key role in the neuropathology of cognitive symptoms. Iclepertin (BI 425809), an inhibitor of glycine transporter-1, enhances glutamatergic signalling by increasing synaptic levels of the *N* -methyl-D-aspartate receptor co-agonist, glycine. A 12-wk, Phase II trial (NCT02832037) in 509 patients (pts) with schizophrenia demonstrated that iclepertin was well tolerated and significantly improved cognition.

**Objectives:**

The Phase III CONNEX programme aims to confirm the efficacy, safety and tolerability of iclepertin in improving cognition and functioning in a larger cohort of pts.

**Methods:**

CONNEX consists of three replicate randomised, double-blind, placebo-controlled parallel-group trials in pts with schizophrenia (NCT04846868, NCT04846881, NCT04860830) currently stable on antipsychotic treatment. Each trial aims to recruit ~586 pts, 18–50 years old, treated with 1–2 antipsychotic medications (≥12 wks on current drug; ≥35 days on current dose prior to treatment), who have functional impairment in day-to-day activities and interact ≥1 hr per wk with a designated study partner. Pts with CI due to developmental, neurological or other disorders, or receiving cognitive remediation therapy within 12 wks prior to screening, will be excluded. Pts will be recruited from 39 countries in Asia, Australia, New Zealand, North and South America and Europe, and randomised 1:1 to receive either oral iclepertin 10 mg (n=293) or placebo (n=293) once daily over 26 wks. The primary efficacy endpoint is change from baseline (CfB) in the MATRICS Consensus Cognitive Battery overall composite T-score. Key secondary efficacy endpoints are CfB in Schizophrenia Cognition Rating Scale total score and CfB in the adjusted total time in the Virtual Reality Functional Capacity Assessment Tool. Long-term safety and tolerability data will be collected in an open-label safety extension study (CONNEX-X).

**Results:**

The studies are currently recruiting (first pts enrolled Aug–Sept 2021), with completion expected in Q2 2024. Here we present an overview of the current study status, including any information relating to screening failures and the experience of collecting these data as part of a large multicountry, multicentre study.

**Conclusions:**

To date, most large, industry-sponsored studies testing various compounds to address cognitive function have failed to show proof-of-clinical concept. Demonstration of efficacy of iclepertin in improving cognition in this Phase III programme would provide important insight into the role of glutamate in cognitive symptoms, that may also have relevance for other cognitive disorders. Iclepertin may represent the first efficacious medication for CI associated with schizophrenia.

**Disclosure of Interest:**

P. Falkai Consultant of: Advistory board Boehringer Ingelheim Pharma GmBH & Co. KG, C. Reuteman-Fowler Employee of: Boehringer Ingelheim Pharma GmBH & Co. KG, Z. Blahova Employee of: Boehringer Ingelheim Pharma GmBH & Co. KG, S. Ikezawa Consultant of: Financial agreements with Boehringer Ingelheim Pharma GmbH, Merck, Biogen and Sunovion, S. Marder Grant / Research support from: Boehringer Ingelheim, GW Pharma, Consultant of: Boehringer Ingelheim, Merck, Sunovion, J. Krystal Shareolder of: Co-founder of Freedom Biosciences, Inc Investments in Biohaven Pharmaceuticals, Sage Pharmaceuticals, Spring Care, Biohaven Pharmaceuticals Medical Sciences, EpiVario, RBNC Therapeutics, Terran Biosciences and Tempero Bio., Consultant of: Aptinyx, Atai Life Sciences, AstraZeneca Pharmaceuticals, Biogen, Biomedisyn Corporation, Bionomics, Boehringer Ingelheim International, Cadent Therapeutics, Clexio Bioscience, COMPASS Pathways, Concert Pharmaceuticals, Epiodyne, EpiVario, Greenwich Biosciences, Heptares Therapeutics, Janssen, Jazz Pharmaceuticals, Otsuka America Pharmaceutical, Perception Neuroscience Holdings, Spring Care, Sunovion Pharmaceuticals, Takeda Industries, Taisho Pharmaceutical Co Advistory board: Biohaven Pharmaceuticals, BioXcel Therapeutics, Cadent Therapeutics, Cerevel Therapeutics, Delix Therapeutics, EpiVario, Eisai, Jazz Pharmaceuticals, Novartis, PsychoGenics, RBNC Therapeutics, Tempero Bio and Terran Biosciences;

